# Ruptured giant splenic artery aneurysm with an exceptional concurrent gastric and transverse colonic fistula: A rare case report

**DOI:** 10.1097/MD.0000000000039159

**Published:** 2024-08-02

**Authors:** Abdo Mohamad Zain, Abdoul Majid Sires, Mohammad Al-Jawad, Hussein Alkanj

**Affiliations:** a Department of Vascular Surgery, Faculty of Medicine, University of Aleppo, Aleppo, Syrian Arab Republic; b Faculty of Medicine, University of Aleppo, Aleppo, Syrian Arab Republic.

**Keywords:** colon perforation, fistula, gastric perforation, splenic artery aneurysm, thoracotomy

## Abstract

**Introduction:**

Splenic artery aneurysm (SAA) is a focal dilation of the splenic artery with varying etiologies including atherosclerosis, arteritis, or trauma. Giant SAAs with a diameter of 10 cm is rare and can lead to severe complications like rupture and fistulas. Therefore, an accurate and timely diagnosis and treatment are important.

**Patient concerns:**

A 50-year-old male presented with acute epigastric pain and hemorrhagic shock. Considering his symptoms and examination, ultrasound, multi-slice computed tomography and digital subtraction angiography results, a ruptured giant splenic artery aneurysm complicated with an exceptional gastric and transverse colonic fistula was suspected.

**Diagnosis:**

Ruptured giant splenic artery aneurysm.

**Interventions:**

Left anterolateral thoracotomy to control the severe aortic bleeding just above the diaphragm, aneurysmectomy, splenectomy, and closing the gastric and transverse colon perforations.

**Outcomes:**

Multi-slice computed tomography demonstrated the presence of splenic artery aneurysm in the distal third measuring (10 × 12 cm) in diameter with a true lumen measuring (7 × 3.5 cm) and a large hematoma extending to the greater and lesser gastric curvature. Intraoperatively, a large pulsating mass was detected occupying the epigastrium and the left hypochondrium with severe adhesions with the stomach and transverse colon.

**Conclusion:**

Giant SAA with a diameter of 10 cm is rare and is associated with severe complications. Therefore, successful treatment of splenic artery aneurysms involves prompt diagnosis, immediate surgical intervention to control bleeding, and tailored approaches like thoracotomy to control the thoracic aorta for better hemodynamic stabilization, aiming to eliminate the aneurysm and reduce complications effectively.

## 1. Introduction

Splenic artery aneurysm (SAA) is defined as a focal dilation in the diameter of the splenic artery that is 50% greater than the normal vessel diameter.^[1]^ Usually, the pathology concerns elastic fibers and smooth muscle cells of the middle part of the vessel.^[2]^ Histopathologically, SAAs are classified into 2 types: true aneurysm and pseudoaneurysm (PSA). The exact etiology of visceral aneurysms is not established. Recent literature has suggested that true aneurysms develop secondary to arterial wall weakness due to several causes. These include atherosclerosis, medial degeneration or dysplasia, abdominal trauma, hypertension, connective tissue diseases, and necrotizing vasculitis such as polyarteritis nodosa or Wegner granulomatosis. PSAs are periarterial hematomas that develop as a consequence of iatrogenic trauma or inflammatory processes such as chronic pancreatitis. PSAs lack a true wall hence they are more prone to rupture.^[3]^ Although SAAs are relatively rare, they are the most common type among visceral vessel aneurysms, occurring with an incidence ranging from 0.2% to 2%. The annual risk of rupture of a SAA is among 2% and 10%. Notably, SSAs exhibit a higher incidence in females (4:1).^[2]^ Giant splenic artery aneurysm (GSAA) presents at a mean age of 56.1 ± 17.3 years. Abdominal pain is the predominant presenting symptom in over 50% of documented cases.^[4]^ Additionally, patients may present with other symptoms such as hematochezia, melena, hematemesis, or hemosuccus pancreaticus.^[5]^ True aneurysms are usually asymptomatic, in contrast to PSAs, which have a reported rupture rate ranging from 37% to 47% and can lead to severe consequences. Rupture of SAAs is typically detected as bleeding into the lesser sac, peritoneal space, and adjacent areas.^[6]^ Fistulation with vascular structures like the splenic and portal veins can cause arteriovenous fistulae. These fistulae can cause a mesenteric steal phenomenon, resulting in insufficient blood supply to the small bowel and subsequent ischemia. Furthermore, the external mass effect exerted on the portal vein can lead to portal hypertension and venous congestion. Additionally, SAAs have the potential to rupture into pancreatic pseudocysts.^[7]^ The diagnostic process primarily relies on contrast-enhanced computed tomography scans, enabling visualization of the lesions during the arterial phase. Doppler ultrasound and magnetic resonance imaging also serve as crucial supplementary modalities for accurate diagnosis. However, digital subtraction angiography or transcatheter angiography via the femoral artery is regarded as the gold standard investigation due to its therapeutic capabilities.^[5]^ The most commonly performed procedure is aneurysmectomy and splenectomy with or without additional resection. Overall, surgical treatment has a lower morbidity than endovascular therapy and comparable reintervention and mortality rates.^[4]^

We report the case of a patient presented with an emergent severe epigastric pain and hemorrhagic shock, who was found to have a ruptured GSAA. He was successfully treated using surgical approach. This case report is, to our knowledge, the first case of a GSAA complicated with a fistula to the stomach and transverse colon simultaneously. It is important to highlight that the patient initially presented with rare symptoms and complications. We highlight the challenges encountered in managing this unique case as a per the SCARE 2023 Guidelines.^[8]^

## 2. Case presentation

A 50-year-old male presented to the ER with acute epigastric abdominal pain, dizziness, and altered consciousness accompanied with a hemorrhagic shock (blood pressure of 70/40 mm Hg, hemoglobin of 3.3 g/dL). The patient is a heavy smoker without medical, surgical, or familial past history. In addition to that the patient complained of early satiety after meals and had a history of 20 kg weight loss during the last 7 months prior to the current admission despite having good appetite.

**Figure 1.**
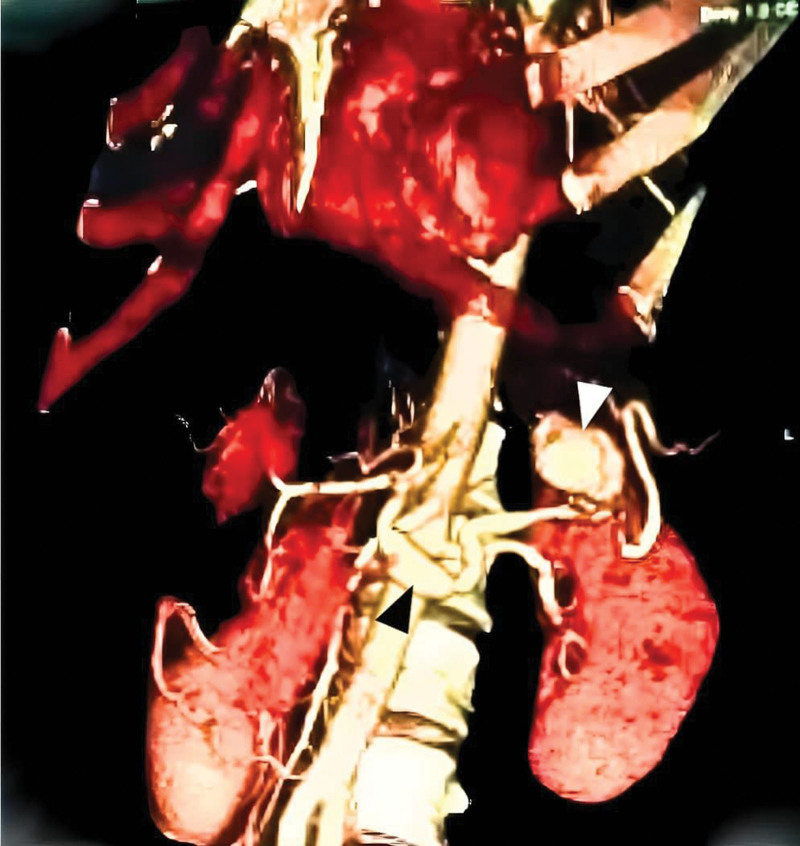
MSCT with three-dimensional reconstruction showing a SAA in the distal part of splenic artery (white arrow) which was done 7 months before current admission. Another aneurysmal dilatation in the first part of splenic artery is revealed (black arrow). MSCT = multi-slice computed tomography, SAA = splenic artery aneurysm.

**Figure 2.**
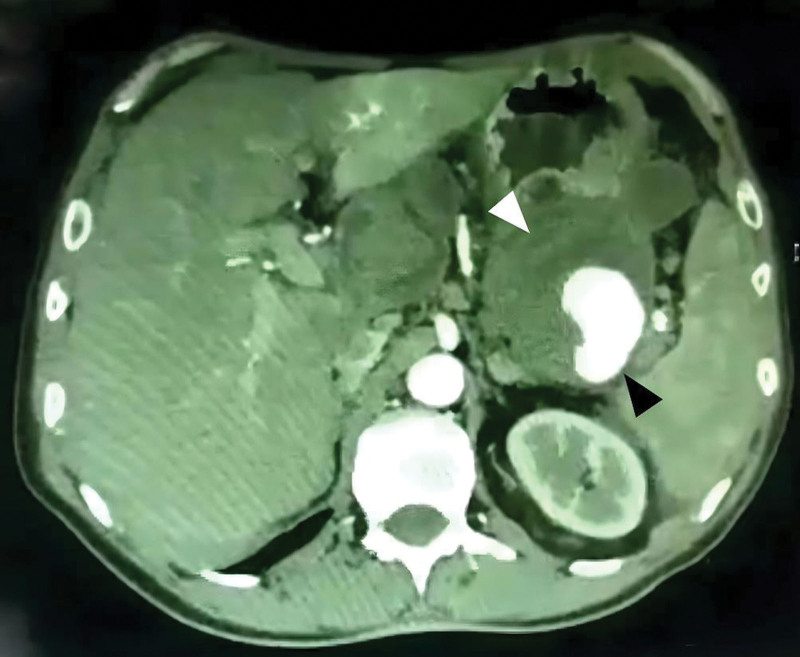
MSCT (cross-sectional image) shows a giant SAA with eccentric thrombosis (white arrow) and a true lumen (black arrow). MSCT = multi-slice computed tomography, SAA = splenic artery aneurysm.

His clinical history began 7 months ago when he suffered suddenly from acute epigastric pain followed after an hour by loss of consciousness. The patient was referred to the hospital and on that admission; he was pale with hemodynamic collapse state. The initial lab test included a hemoglobin of 5.1 g/dL, an international normalized ratio of 1.4, and the fecal occult blood test was negative. The patient was resuscitated by blood transfusion. Several investigations were done as follows: abdominal ultrasound revealed an arterial aneurysm in the epigastric region measuring (9 × 11 cm) with a large hematoma surrounding it. Then upper GI endoscopy was done revealing a small sliding diaphragmatic hernia without presence of peptic ulcers. After that contrast-enhanced multi-slice CT (MSCT) demonstrated the presence of splenic artery aneurysm in the distal third measuring (10 × 12 cm) in diameter with a true lumen measuring (7 × 3.5 cm) (Figs. 1 and 2) and a large hematoma extending to the greater and lesser gastric curvature. The other abdominal organs and viscera were normal. Despite all these investigations, the patient did not complete his treatment because he had rejected the operation at that time. During the last month prior to current admission, the patient complained of fever, chills, and sweating without other complaints. The symptoms were treated with antibiotics and resolved within a week.

Last week before current admission, the patient suffered from hematemesis and melena on 2 occasions and hematochezia for one time. In the current admission, abdominal ultrasound showed an epigastric aneurysm with a large hematoma surrounding it measuring (9 × 10 cm) and another hematoma behind the left kidney measuring about (12 × 17 cm). Despite all resuscitation attempts, the hemodynamic collapse persisted.

**Figure 3.**
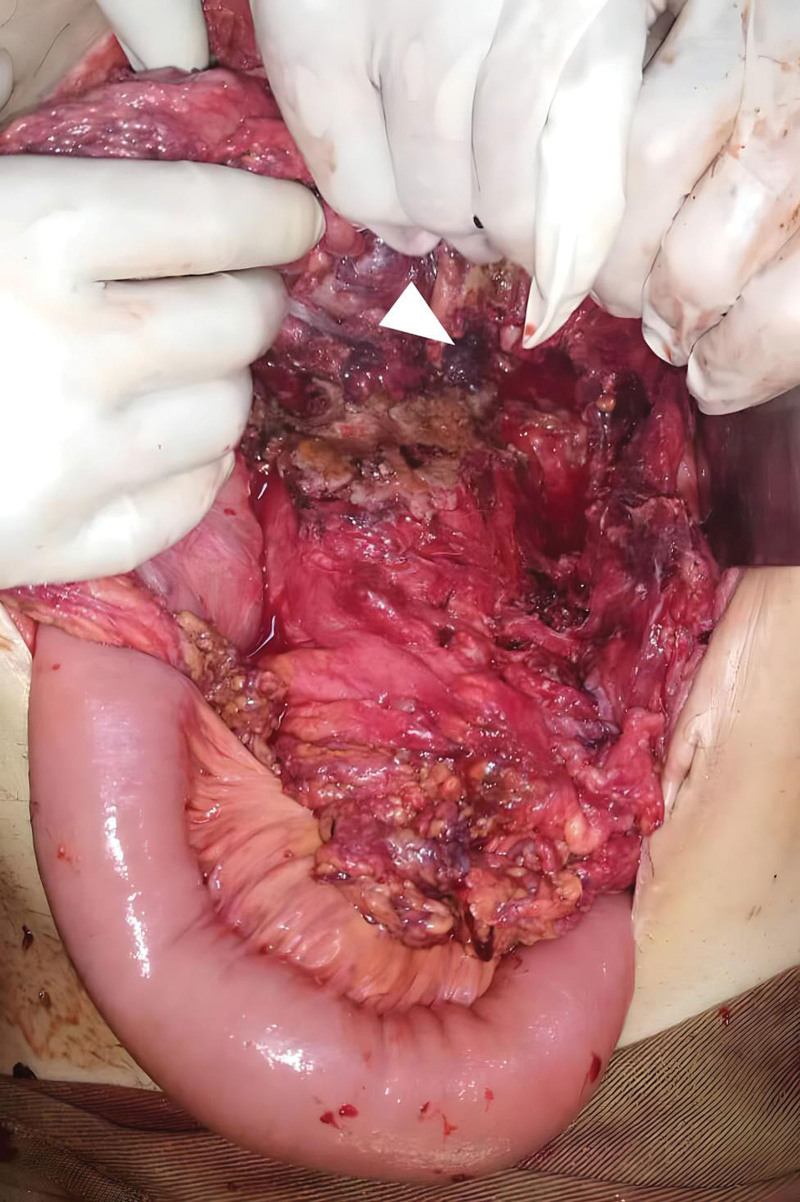
Intraoperative image demonstrating a gastric perforation (white arrow) with adjacent fibrosis due to necrosis of the gastric wall resulting from chronic pressure of hematoma.

**Figure 4.**
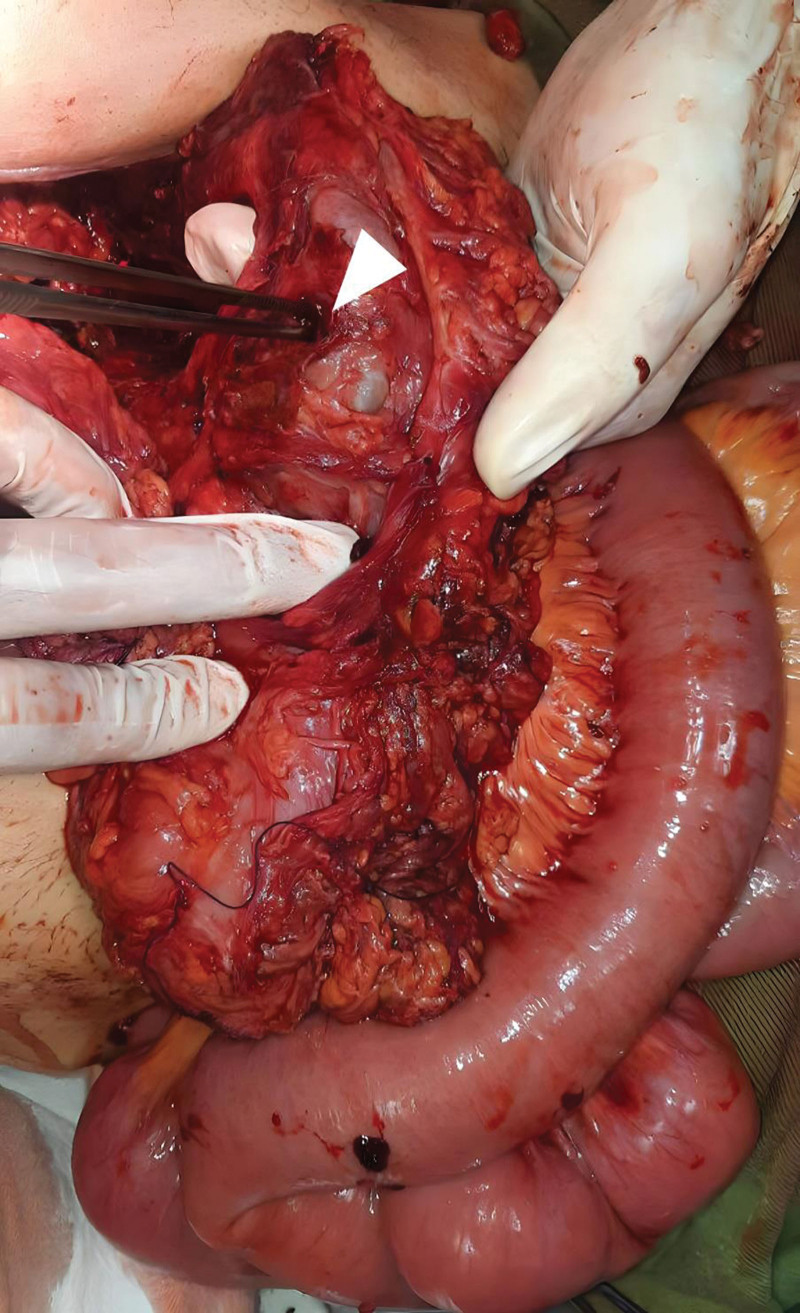
Intraoperative image demonstrating the transverse colon fistula (white arrow).

So, an exploratory laparotomy without any further investigations was done, due to the life-threatening condition. A midline laparotomy incision was performed. A large pulsating mass was detected occupying the epigastrium and the left hypochondrium with severe adhesions between the stomach, colon, omentum, and the pulsating mass. Due to the inability to control the subdiaphragmatic aorta, we resorted to a left anterolateral thoracotomy to control the thoracic aorta just above the diaphragm. After entering the lesser sac, a purulent collection mixed with clots; hiding a gastric perforation on the posterior gastric wall and a transverse colon perforation with fibrous edges, was detected (Figs. 3 and 4). Then the aneurysm was opened and large amounts of clots were removed until we exposed the splenic artery defect. The splenic artery and vein were ligated, the stomach and colon perforation were closed, then splenectomy was performed. During the surgical exposure, it was detected that the omentum was stained with bile, which indicates an old leakage mostly resulted from the compressive effect of the aneurysm and the large hematoma surrounding it on the duodenum leading to bile passage from the gastric perforation.

Postoperatively, the patient developed number of complications including, right pneumothorax mostly resulted from central venous line insertion which was treated by thoracostomy, atelectasis of the left lung which was managed successfully by bronchoscopy, pancreatic fistula and bile leakage which recovered spontaneously. Fortunately, clamping the thoracic aorta did not cause any neurological deficit.

**Figure 5.**
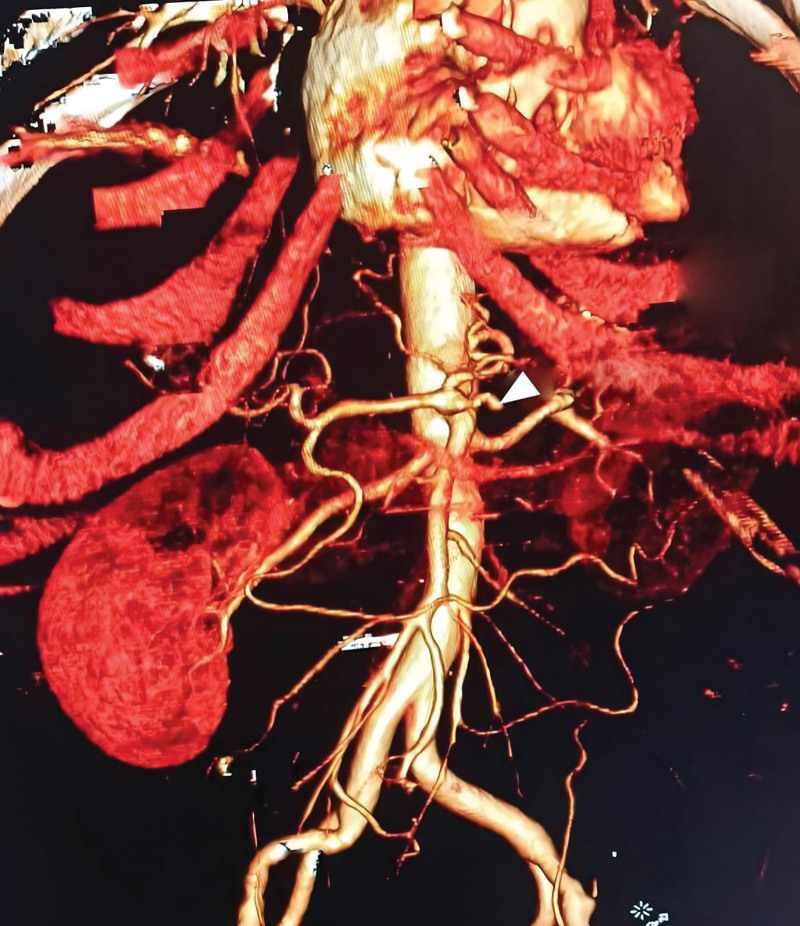
Follow-up three-dimensional reconstruction MSCT demonstrates the splenic artery stump (white arrow) with the absence of any other visceral aneurysm. MSCT = multi-slice computed tomography.

MSCT scan was performed after surgery to evaluate the results of the operation, as shown in Fig. 5, and the patient was discharged on the 13th postoperative day.

## 3. Discussion

The size distribution of SAAs varies, with the majority measuring <2 cm (60% of cases), predominantly affecting the middle or distal portions of the artery.^[9]^ In rare instances, SAAs can reach giant proportions, and in our reported case it measured (10 × 12 cm) in diameter.

Most small SAAs are typically asymptomatic, accounting for approximately 75% to 80% of cases. However, in instances where symptoms do arise, they often manifest with nonspecific features, which can make diagnosis challenging. For example, Invasion into the stomach, duodenum, pancreatic duct, and colon may lead to gastrointestinal bleeding. It has been reported that approximately 13% of ruptured splenic artery aneurysms are associated with fistulas involving these structures.^[7]^ Furthermore, rupture of a SAA with erosion into the stomach is a rare cause of massive upper gastrointestinal bleeding. The gastric fistula of a true giant SAA is even more rare and usually leads to death.^[2]^ Also, colonic fistula occurrence is extremely rare, and the first 2 non-fatal cases of SAA with colonic fistula were reported in 1984 and 2003.^[7]^

In our case, the patient suffered from weight loss that was explained by the satiety resulting from the compressive effect of the aneurysm and surrounding hematoma on the stomach. Also, the signs and symptoms of infection that the patient reported 1 month before the current admission are attributed to the abscess which developed secondary to the gastric perforation.

The patient had a fistulation into the stomach and transverse colon simultaneously, each one of them in itself is a rare complication of SAA, and their co-occurrence together, as far as we know, is the first case reported in the medical literature.

However, various radiological modalities can aid in the identification of SAAs. Digital subtraction angiography is considered the gold standard due to its ability to provide simultaneous diagnosis and intervention. Other imaging options include ultrasound with Doppler, computed tomography (CT) with or without angiography, and magnetic resonance imaging with or without angiography. The choice of modality depends on resource availability and patient factors such as concurrent renal dysfunction or pregnancy, which are a major risk factor for SAAs.^[10]^

The choice of treatment for SAAs is influenced by several factors, including age, sex, aneurysm size, location, complications, and the severity of clinical manifestations. Common treatment options include open abdominal surgery, endovascular procedures (such as coil embolization or stent placement), laparoscopic surgery (which is gaining popularity), and medical management.^[4]^

Recently released guidelines state that SAAs <3 cm in size which are asymptomatic and show little to no growth can be safely observed and monitored with serial imaging. The overall consensus in the literature is that asymptomatic SAA >3 cm in diameter or with interval growth of >0.5 cm/year should be treated.^[9]^

Due to the absence of any clinical or radiologic evidence of acute or chronic pancreatic disease, other inflammatory processes near the splenic artery, or iatrogenic trauma, we lean towards advocating that this case is a true aneurysm.

Surgical intervention has been frequently reported as the primary treatment approach for gastric fistula of a true GSAA, demonstrating an overall success rate comparable to the recent series (98%). This particular approach offers significant benefits in cases where the aneurysm causes symptoms due to mass effect, hemodynamic instability, arteriovenous fistula, or if there are concomitant visceral aneurysms. Surgical intervention is also recommended for aneurysms larger than 10 cm, infected aneurysms, and distal lesions, as it helps mitigate the potential risk of end-organ ischemia that may arise after endovascular embolization.^[4]^

Open abdominal surgery remains the established standard for SAA treatment, despite advancements in techniques. Aneurysmectomy with end-to-end reconstruction is typically preferred for proximally located, elongated, and tortuous SAAs. This approach aims to preserve the spleen, an essential component of the immune system.^[3]^

Resection of an aneurysm can be combined with splenectomy in cases of a hostile abdomen or distal aneurysms located near the splenic hilum.

So, we believed that splenectomy was a valuable procedure to accomplish the control of the bleeding in this patient rather than a splenic tissue-preserving procedure.

In cases involving giant SAAs or when simple aneurysmectomy is unfeasible due to significant structures, alternative surgical options are considered. These may include aneurysmectomy combined with splenectomy, bipolar splenic artery ligation with or without aneurysmectomy, trans aneurysmal splenic artery ligation, and when required, distal pancreatectomy. The morbidity and mortality rates associated with open abdominal surgical interventions for SAAs are reported as 9% and 1.3%, respectively.^[7]^

In case of severe uncontrollable intraabdominal bleeding like our case, a left anterolateral thoracotomy at the level of 7th intercostal space is recommended to control the aorta just above the diaphragm and then be able to remove the aneurysm.

It is important to assess each patient individually and consider their specific circumstances to determine the most appropriate treatment approach for SAAs, taking into account potential risks and benefits associated with each option.

## 4. Conclusion

Achieving successful treatment outcomes for SAAs relies on maintaining a high level of suspicion during diagnostics, conducting thorough angiographic studies, and implementing appropriate surgical or interventional radiological interventions. The primary objective is to effectively occlude or eliminate the aneurysm, thereby mitigating the risk of bleeding.

In certain situations, such as ours, performing immediate laparotomy to control bleeding is more critical than wasting time attempting to do MSCT, to avoid losing precious minutes crucial for saving a life.

In cases of severe intraoperative bleeding, without ability to isolate and control the abdominal aorta, and when endovascular intervention is unavailable, a left anterolateral thoracotomy may be employed to gain access to and control the thoracic aorta. This approach aims to minimize excessive blood loss and prevent further hemodynamic compromise until definitive treatment of the aneurysm can be initiated.

By employing these approaches, the aim is to eliminate the aneurysm and associated risks, ultimately leading to successful treatment outcomes and minimizing the potential for further complications.

## Author contributions

**Conceptualization:** Abdo Mohamad Zain, Mohammad Al-Jawad, Hussein Alkanj, Abdoul Majid Sires.

**Data curation:** Hussein Alkanj, Abdo Mohamad Zain.

**Investigation:** Abdo Mohamad Zain, Hussein Alkanj.

**Methodology:** Abdo Mohamad Zain, Hussein Alkanj.

**Project administration:** Abdo Mohamad Zain, Hussein Alkanj.

**Resources:** Abdo Mohamad Zain, Abdoul Majid Sires, HusseinAlkanj, Mohammad Al-Jawad.

**Software:** Hussein Alkanj.

**Supervision:** Abdo Mohamad Zain, Hussein Alkanj.

**Validation:** Abdo Mohammad Zain, Abdoul Majid Sires, Mohammad Al-Jawad, Hussein Alkanj.

**Visualization:** Abdo Mohammad Zain, Abdoul Majid Sires, Mohammad Al-Jawad, Hussein Alkanj.

**Writing – original draft:** Abdoul Majid Sires, Mohammad Al-Jawad.

**Writing – review & editing:** Abdoul Majid Sires, Mohammad Al-Jawad.
